# A Case of Advanced Extramammary Paget’s Disease With a High Tumor Mutation Burden That Showed Partial Lymph Node Regression With Pembrolizumab

**DOI:** 10.7759/cureus.72592

**Published:** 2024-10-28

**Authors:** Ken Horisaki, Tomoki Taki, Shoichiro Mori, Mao Okumura, Masashi Akiyama

**Affiliations:** 1 Dermatology, Nagoya University, Nagoya, JPN

**Keywords:** empd, extramammary paget’s disease, pembrolizumab, tmb-high, tumor mutation burden (tmb)

## Abstract

Pembrolizumab has been found effective against various solid tumors with high tumor mutation burden, but there are no reports of successful treatment with pembrolizumab for extramammary Paget's disease (EMPD) with a high tumor mutation burden (TMB). This report describes a 71-year-old male patient who presented with irregularly shaped erythematous lesions on his scrotum, which had been there for several years. He was diagnosed with EMPD. A gene panel test indicated a high TMB. Six months after radical surgery, many lymph node metastases were found. We started a combination chemotherapy of tegafur-gimeracil-oteracil potassium (TS-1) and docetaxel. After three courses, a CT scan showed reductions in the sizes of all the metastatic lesions. However, the treatment was switched to pembrolizumab at the patient’s request. After four cycles of the pembrolizumab treatment, a CT scan showed further shrinkage in most of the lymph node metastases. As far as we know, the present patient is the first case of advanced EMPD with response to pembrolizumab monotherapy.

## Introduction

Extramammary Paget’s disease (EMPD) is a rare malignant neoplasm that is commonly localized to apocrine gland-rich skin. Due to its low incidence, there is little specific evidence on systemic therapies for EMPD. A recent report found pembrolizumab, an immune checkpoint inhibitor (ICI), to be effective against various solid tumors with high tumor mutation burdens (TMBs), but there are no reports of successful treatment with pembrolizumab for EMPD with a high TMB [1]. As far as we know, the present patient is the first case of advanced EMPD with response to ICI monotherapy.

## Case presentation

**Figure 1:**
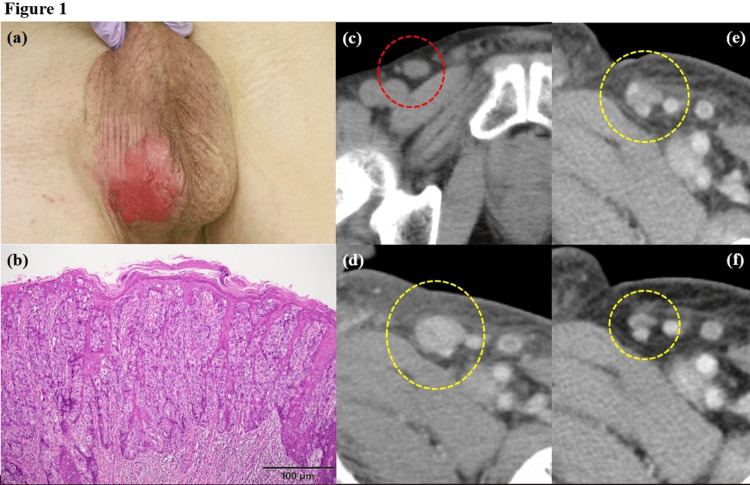
Clinical and histopathological features and computed tomographic (CT) images of the present patient. (a) The primary lesion at initial examination: erythematous lesions with several nodules and erosions with marginal irregularities on the right side of the scrotum. (b) Histopathology of a skin biopsy at the initial examination: the prominent proliferation of Paget cells in the lesional epidermis. (c) A CT image at the initial examination. CT scan showed one enlarged lymph node in the right inguinal region (the red dotted circle). (d) A CT image before starting docetaxel and TS-1 combination therapy. Enlargement of lymph nodes, including the left inguinal (the dotted yellow circle), bilateral external and internal iliac, the para-aortic, the left subclavian, and the bilateral paratracheal and subtracheal lymph nodes, was observed. (e) A CT image after three courses of docetaxel and TS-1 combination therapy, before starting pembrolizumab. The dotted yellow circle indicates the left inguinal lymph node. (f) A CT image after four cycles of pembrolizumab. Most of the lymph nodes, including the left inguinal (the dotted yellow circle) have shrunk.

A 71-year-old male patient was referred to our clinic with irregularly shaped erythematous lesions on the scrotum that had been present for several years (Figure 1a). A skin biopsy revealed the focal proliferation of Paget cells within the epidermis and their microinvasion into the dermis, leading to the diagnosis of primary EMPD (Figure 1b). No other special staining or immunostaining was performed. A computed tomography (CT) scan showed one enlarged lymph node in the right inguinal region (Figure 1c). As the initial treatment, wide local excision of the primary tumor with a 1 cm margin and right inguinal and pelvic lymph node dissection were performed, and metastasis was found in the inguinal lymph node (1/7) and in the pelvic lymph nodes (5/12). A CT scan six months after the surgery suggested multiple metastases to the following lymph nodes: the left inguinal, bilateral external and internal iliac; the para-aortic; the left subclavian; and the bilateral paratracheal and subtracheal (Figure 1d).

We started the combination chemotherapy of tegafur-gimeracil-oteracil potassium (TS-1) (120 mg/m2 per day for 14 consecutive days) and docetaxel (40 mg/m2 per day on day one) at four-week intervals. After three courses, a CT scan showed reductions in the sizes of all the metastatic lesions (Figure 1e). A gene panel test (Foundation One CDx; Foundation Medicine, Inc., Cambridge, Massachusetts, United States) identified multiple genomic alterations in genes including ARID1A, INPP4B, MYC, RAD21, SOX9, and TP53, and indicated a high TMB of 13.0 mutations per megabase and microsatellite stable. The patient achieved a partial response (PR) to the docetaxel and TS-1 combination therapy, but treatment was switched to pembrolizumab (200 mg/body, every three weeks) at the patient’s request. A CT scan after four cycles of pembrolizumab showed further shrinkage in most of the lymph node metastases with an overall reduction rate of 30% compared to before pembrolizumab administration (Figure 1f). However, a CT scan after a further five cycles showed multiple enlarged lymph nodes, including newly found liver metastasis and peritoneal dissemination. Four courses of docetaxel and TS-1 combination therapy followed, but the tumor progressed further, and he eventually died approximately two years after the diagnosis of EMPD.

## Discussion

EMPD typically grows slowly, and cases diagnosed as an in situ lesion generally show favorable prognosis following surgical resection. However, once it invades the dermis, EMPD is prone to metastasize, resulting in a poor prognosis [2]. In the present case, multiple metastases were found after the wide local resection of the primary lesion. We speculate that at the time of the wide local excision, there were already micro-metastases in multiple lymph nodes. Due to the rarity of EMPD, few clinical trials of systemic therapies for EMPD have been conducted [3]. However, in other solid tumors with high TMB, one study on the efficacy of ICIs showed that high TMB may be a useful biomarker for predicting the response to pembrolizumab monotherapy in patients with advanced solid tumors [1]. The median progression-free survival (PFS) in the study was 2.1 months (95% CI: 2.1-4.1), but some of the patients showed long-term improvement in overall survival [3].

In the present case, we expected long-term improvement, but the PFS was six months. Two studies have addressed ICI monotherapies for advanced EMPD with high TMB, but neither reported a successful response [4,5]. In the first case, pembrolizumab was administered for EMPD as the first-line treatment, but after six cycles it was determined to be a progressive disease [4]. In the second case, pembrolizumab was given for three cycles as the third-line treatment after the use of two cytotoxic chemotherapies, but the efficacy assessment showed a progressive disease [5]. In contrast, the present case is unique in that treatment was switched to pembrolizumab while the previous therapy was still effective. That situation, together with the high TMB, may have contributed to the response. However, even in this case, the effect of pembrolizumab was temporary, and its effect was not sustained.

Regarding the safety of pembrolizumab, a retrospective study of pembrolizumab monotherapy in 8973 patients has been reported [6]. Any-cause adverse events (AEs) were reported to have occurred in 96.6% of patients (grade 3-5 AEs, 50.6%), with immune-related adverse events (irAEs) and infusion reactions occurring in 23.7%. The most common AEs of any cause were fatigue (29.7%), nausea (20.4%), and decreased appetite (20.3%), and grade 3 or higher AEs occurred in 50.6% of patients, with anemia (5.4%), pneumonia (4.0%), hyponatremia (2.7%), fatigue (2.6%), and dyspnea (2.1%) being the most common. Fortunately, the patient had minor fatigue and loss of appetite, but no major AEs in the present case.

## Conclusions

In this report, we describe a valuable case of EMPD with a high TMB that responded to pembrolizumab monotherapy. Due to its rarity, few clinical trials of systemic therapies for EMPD have been conducted, and sufficient evidence for systemic treatment of EMPD has not been obtained yet. In this case, pembrolizumab was temporarily effective, but its effect was not sustained. A further accumulation of cases is needed to evaluate the effectiveness of ICI monotherapies for EMPD.

## References

[1] Tanese K, Nakamura Y, Hirai I, Funakoshi T (2019). Updates on the systemic treatment of advanced non-melanoma skin cancer. Front Med (Lausanne).

[2] Kibbi N, Owen JL, Worley B (2022). Evidence-based clinical practice guidelines for extramammary Paget disease. JAMA Oncol.

[3] Marabelle A, Fakih M, Lopez J (2020). Association of tumour mutational burden with outcomes in patients with advanced solid tumours treated with pembrolizumab: prospective biomarker analysis of the multicohort, open-label, phase 2 KEYNOTE-158 study. Lancet Oncol.

[4] Nakayama Y, Ogata D, Wada S (2023). Treatment of high tumor mutation burden metastatic extramammary Paget disease with an anti-PD-1 antibody. J Dermatol.

[5] Koizumi S, Doi R, Aitake U (2024). Unfavorable efficacy of pembrolizumab for advanced extramammary Paget's disease with high tumor mutation burden after failure of taxane-based regimens. J Dermatol.

[6] Brahmer JR, Long GV, Hamid O (2024). Safety profile of pembrolizumab monotherapy based on an aggregate safety evaluation of 8937 patients. Eur J Cancer.

